# Providing Tech Support as Care Work Among Care Workers in Assisted Living Facilities: Qualitative Interview Study

**DOI:** 10.2196/80272

**Published:** 2026-02-26

**Authors:** Yiyi Wu, Nicole Ross, Sunyoung Kim

**Affiliations:** 1 School of Communication and Information Rutgers, The State University of New Jersey New Brunswick, NJ United States; 2 Brookdale Center for Healthy Aging Hunter College New York, NY United States

**Keywords:** assisted living, care workers, long-term care, older adults, technology use, technology support

## Abstract

**Background:**

Technology can enhance the quality of life and social engagement for older adults; yet, many require assistance to use it effectively. In assisted living facilities, care workers play a crucial role in supporting residents’ use of technology. However, little research has examined the experiences and challenges of care workers in this context.

**Objective:**

In this study, we examine the perspectives and experiences of care workers in supporting older residents with technology.

**Methods:**

We conducted semistructured interviews with 20 professional care workers in the United States, with an average of 4.9 years of experience working in assisted living facilities.

**Results:**

The findings indicate that participants regularly provided technology support to their residents, while their perspectives on and acceptance of this technology support role varied. For many, this responsibility placed additional demands on their existing workload, requiring both explicit and nuanced physical and emotional labor input.

**Conclusions:**

These findings underscore the need for greater recognition and systemic support for care workers as they increasingly take on the responsibility of facilitating technology use in residential care settings.

## Introduction

### Background

Assisted living facilities, a type of long-term care, serve older adults who can no longer live safely and independently at home. These residential communities provide housing and are staffed with care workers who support residents with a wide range of daily needs and activities [1]. Assisted living emphasizes person-centered care within a home-like environment, catering to a broader spectrum of care and social needs, including those of residents with complex medical conditions [2]. This sector represents a fast-growing segment of long-term care in the United States [3]. However, chronic staffing shortages pose ongoing challenges, which are exacerbated by the rapidly growing aging population [4]. In response, digital and emerging technologies have been explored as a promising means to help bridge this caregiving gap [5,6]. Examples include the use of information and communication technologies (ICTs) to facilitate proactive social engagement and health communication, as well as wearable devices for disease self-management [6-8]. These innovations have demonstrated significant potential to enhance the well-being of older adults, improve their quality of life, and reduce caregiver burden, both within long-term care settings and beyond [6,9].

Yet, the successful applications of technology depend on effective usage, and older adults often face barriers due to lower digital skills, physical and cognitive declines, and a lack of prior exposure to technology [10,11]. While the ownership of digital technologies among older adults has grown [12], they continue to struggle with independent use, often experiencing anxiety, distrust, and avoidance of technology [13-15].

Consequently, studies consistently highlight that older adults’ adoption and effective use of technology heavily depend on their immediate support networks, such as their children and community workers, for assistance with technology [16-18]. In assisted-living settings, care workers often serve as a critical source of technology support for older residents [19]. The term “care workers” refers to individuals who provide essential physical, emotional, and practical support to people requiring assistance with activities of daily living due to age, illness, disability, or other life circumstances [20]. This category encompasses a wide range of occupational roles, including nurses, nursing assistants, and care aides [4,20]. Despite variation in professional training and job titles, care workers share core responsibilities centered on facilitating mobility, administering medication, assisting with daily routines, and supporting the overall well-being of care recipients. Collectively, their labor constitutes a critical component of long-term care systems and plays a vital role in maintaining the quality of life of older adults [4,21].

Previous research on technology use in long-term care indicates that as more older residents adopt digital technologies, their reliance on care workers for technological support may also introduce additional work demand and challenges for care workers [21,22]. However, while emerging studies have begun to attend to those challenges [19,21], the literature has given little attention to care workers’ perspectives on and experiences with providing such technology support to older residents. Consequently, there remain limited insights into how to best support care workers within increasingly digitalized caregiving environments.

Understanding the perspectives and experiences of care workers is particularly important given the vulnerabilities they face in the workplace. Caregiving often demands intensive physical and emotional labor, with care workers frequently expected to invest effort beyond their formal job requirements while receiving limited financial compensation and insufficient recognition from employers [21-24]. As the demand for assisted living continues to grow [25], providing better support for care workers is essential for enhancing staff retention and the quality of care.

To explore these issues, we conducted semistructured interviews with 20 care workers employed in assisted living facilities in the United States. Our study aimed to answer the following research questions:

1. How do care workers perceive their involvement in providing technology support?
2. What challenges do care workers face as they navigate the provision of technology support?

Our findings indicate that, although participants held varying views on their engagement in technology support, they consistently recognized it as an integral part of care workers’ daily responsibilities. This role adds new dimensions of physical and emotional labor beyond their core job duties. Moreover, the heavy reliance of older residents on care workers for tech support highlights the need for greater recognition of this labor, as well as involving both care workers and older adults in the design of technology for older adults. Additionally, our findings highlight the importance of support programs that empower care workers, enabling them to establish clear work boundaries and navigate their roles more effectively in long-term care settings.

### Literature Review

#### Care Workers’ Roles and Workload

Care workers in long-term care broadly refer to individuals who deliver direct care services to people needing daily assistance, typically older adults, in home or residential settings [26]. In residential long-term care facilities, such as assisted living, these professionals are commonly referred to as care assistants, social workers, nursing assistants, and other health care professionals [20,27]. Prior studies indicate that care workers’ roles and job responsibilities are often loosely defined, primarily determined by the individualized needs of their residents [28]. This role ambiguity is particularly pronounced in residential care settings, where person-centered care is a key emphasis [29,30]. While fostering meaningful relationships with residents is central to care work, this closeness can blur professional boundaries, making it difficult to delineate responsibilities [29,30]. As a result, care workers frequently feel an overarching sense of duty for residents’ well-being, taking on multiple roles beyond their formal job descriptions [28,30]. Qualitative studies on care workers’ attitudes toward their responsibility further highlight a persistent tension between care workers’ commitment to holistic resident care and the emotional strain of multitasking, burnout, and heavy workloads [23,31].

Commonly recognized care workers’ routine responsibilities include, but are not limited to, hygiene assistance, medication management, mobility support, case management, and administrative tasks [32]. While these duties already demonstrate the labor-intensive nature of caregiving, research further reveals that many essential caretaking roles and tasks remain unseen and unpaid [23,28]. These include the emotional investment involved in building trust relationships with clients, managing their clients’ emotional responses, and regulating one's own emotions in high-stress environments [33,34]. Additionally, care workers often coordinate to facilitate communication between their clients, health care providers, and other third parties [19,28]. Ongoing studies have continuously advocated for greater recognition of care workers' contribution, particularly the less recognized aspects of their labor, to improve their overall financial and social well-being [23,29].

#### Technology Use Among Older Adults in Long-Term Care

A growing body of literature has explored the role of technology in enhancing the quality of life and supporting independent engagement in social and health management activities among older adults in long-term care settings [7,9,35]. ICTs, such as computers, laptops, smartphones, and mobile apps, play a crucial role in enabling older residents to maintain connections with family and friends outside of facilities and engage in various leisure activities [7,36,37]. The use of social media and videoconferencing platforms has been shown to effectively reduce social isolation and feelings of loneliness [37-39]. Beyond social engagement, ICTs are also widely used to deliver essential care services, such as telemedicine and mobile health (mHealth) applications, which facilitate the remote provision of routine care and health information [40,41]. These applications help deliver effective personalized care [40]. Moreover, technologies gaining popularity in long-term care facilities also include wearable devices, such as smartwatches and fitness trackers [6,42], as well as assistive technologies, such as autonomous wheelchairs, designed to enhance functional capacities [43]. These tools support mobility, help monitor health indicators, promote physical activity, and support self-management of chronic diseases [40-43].

The growing use of technologies within long-term care facilities is partly driven by older adults’ voluntary adoption, as evidenced by the steady increase in personal device ownership within this demographic [12]. Additionally, as the long-term care sector continues to experience staffing shortages, these technologies are increasingly recognized for their potential to bridge workforce gaps [44]. Consequently, many facilities have expanded their technological resources by providing residents with access to ICT, assistive, and wearable devices, while actively promoting their use [9,17,37,45]. In some cases, technologies have become so embedded in facility operations that residents must engage with them to carry out routine activities and access services and information within the facility [45]. This trend underscores the increasing significance of technology in the daily lives of older residents in long-term care.

#### Dependency, Technology Support, and Potential Challenges

While most technologies require the direct involvement of older adults, studies have highlighted the heightened challenges that older users face in realizing the intended benefits of these tools [18,46]. Older adults exhibit wide variation in digital literacy, prior experience with technology, and physical or cognitive abilities, all of which affect their capacity to use digital tools independently [13,14,46]. Despite the growing availability of technological resources in long-term care facilities, individuals with higher levels of technological proficiency or prior experience are more likely to engage with these tools [17]. In contrast, those with limited experience or skills often remain disengaged, even when they recognize the potential benefits of technology use [16,17]. Moreover, regardless of their skill levels and motivation, many older adults still struggle with basic interactions due to age-related declines in vision, hearing, fine motor skills, or cognitive processing [21,36]. The continuous diversification of devices and interfaces, each requiring different levels of engagement, from basic device operation to security credential management, can further exacerbate the distrust and anxiety associated with technology use for older adults with cognitive and functional impairments [14,15,46].

Consequently, older adults often rely on close social networks for assistance in using technology effectively [16,18]. These networks typically include family caregivers or other younger family and community members who live in close proximity to older adults [16-18]. In long-term care facilities, several studies have shown that professional care workers often become the primary source of technology support for older residents [19,21]. Nevertheless, providing technology support can be challenging [21,22]. For instance, Hardy et al [22] found that facilitating videoconferencing for cognitively impaired older residents was physically demanding, requiring multiple steps to connect them to virtual platforms. Similarly, Chew et al [21] reported dissatisfaction was found among care workers tasked with setting up and managing virtual visits during the COVID-19 pandemic, a responsibility they found burdensome due to visitor restrictions that placed greater reliance on digital communication.

Despite increasing integration of technology in long-term care facilities, limited research has specifically examined care workers’ experiences with providing technology support to their older residents. While prior research emphasizes the importance of care workers in daily resident support, little is known about how technology-related responsibilities intersect with their existing workloads. This gap in the literature risks rendering this emerging aspect of care work unrecognized, thereby negatively affecting their financial and social well-being [22,23]. By examining the direct experiences and perspectives of care workers, this study aims to inform strategies that better support them in long-term care settings.

## Methods

### Participants

We conducted semistructured interviews with 20 individuals working in assisted living facilities in the Greater New York area. Participants were eligible if they had worked in any care worker role at an assisted living facility for more than 1 year and were at least 18 years old. To recruit participants, we contacted assisted living facilities in the region and provided managers with a detailed explanation of the study’s objectives. Upon their approval, recruitment flyers were distributed via email, and managers and supervisors referred potential participants to the research team.

The final sample included 20 participants (9 male and 11 female), with an average of 4.9 (SD 3.08) years of work experience and an age range of 23 to 56 (mean 38.15, SD 8.23) years. All participants were paid professional care workers employed by an assisted-living facility at the time of the interviews. While the resident base in these facilities exhibits varying levels of independence and diverse health needs, many live with chronic conditions, including diabetes, hypertension, or heart disease, and require mobility support, including assistance with walking and using mobility aids. Some also require monitoring for safety or adherence to care routines.

All participants’ job titles were self-reported. Nine participants identified as caregivers or care assistants; 6 held medical-care-oriented roles, such as nurse or medication assistant, and 4 worked in social-care-oriented roles, such as case managers and activity assistants. One participant held an administrative role but still reported frequent direct interactions and support for residents. A detailed breakdown of participant demographics is provided in Table 1. Job descriptions are synthesized from participants’ accounts and sources, including professional associations such as the American Nurses Association, government websites like the U.S. Department of Health and Human Services, and career websites like Glassdoor. While they offer a general understanding of each role, they may not fully capture the daily experiences of care workers, a discrepancy explored in the Results section.

**Table 1:** Participant demographics.

ID	Gender	Age (years)	Work experience (years)	Job title
P1	Female	48	5	Case Manager^a^
P2	Female	40	8	Case Manager
P3	Female	56	10.5	Case Manager
P4	Male	39	3	Caregiver^b^
P5	Male	33	6	Caregiver
P6	Female	39	10	Caregiver
P7	Female	32	3	Care Assistant^b^
P8	Female	35	2	Care Assistant
P9	Female	32	4	Care Assistant
P10	Female	30	3	Care Assistant
P11	Male	23	3	Care Assistant
P12	Female	29	2	Care Assistant
P13	Male	38	7	Nurse^c^
P14	Female	47	1.5	Nurse
P15	Female	51	1	Nurse
P16	Male	40	8	Nurse
P17	Female	30	1	Nursing Assistant^d^
P18	Male	37	5	Medication Assistant^e^
P19	Male	35	7	Activity Assistant^f^
P20	Female	49	8	Administrator^g^

^a^Case managers are health care professionals who help clients with health management, service coordination, and patient advocacy.

^b^Care assistants, caregivers, or personal care aides are care professionals who provide a broad spectrum of services to those who need assistance with daily activities. Their duties include personal care, domestic care, and companionship.

^c^Nurse is a health care professional who attends to patients’ biological, physical, and behavioral needs. In the United States, all nurses are required to complete formal training to obtain licenses and certifications.

^d^Nurse assistants provide support in daily activities, health monitoring, and hygiene for clients. Different from nurses, nursing assistants are not always certified.

^e^Medication assistant helps administer medications in a hospital or other care facility.

^f^An activities assistant helps to plan events for socialization and enjoyment in a variety of settings.

^g^Long-term care administrator plans, organizes, directs, and manages the operation and implements policies.

### Data Collection

An interview guide was developed to facilitate open-ended, semistructured interviews (Multimedia Appendix 1). The guide consisted of several key topics, with the first few questions concerning participants' job titles and responsibilities, as well as their day-to-day work life. The latter part of the questions focused on participants’ perceptions of their residents’ technology use and experiences helping their residents with technology-related inquiries. This set of questions is followed by those concerning their use of technology. All interviews were conducted virtually using the videoconferencing software of the participants’ choice (eg, Zoom). Each session lasted up to 60 minutes, allowing for an in-depth discussion of their experiences. With participants’ consent, all interviews were audio-recorded and transcribed using transcription software. Each transcript was then carefully reviewed and manually corrected to ensure accuracy.

### Data Analysis

We used a grounded theory approach to identify recurring patterns within our interview data [47]. The process began with open coding, where transcripts were systematically examined to label key concepts related to care workers’ perspectives and experiences with tech support [48]. We then conducted axial coding, organizing related concepts into broader categories, referred to as phenomena, such as recurring events, actions, or interactions that illustrate how care workers navigate their tech support roles. Lastly, we engaged in selective coding to synthesize the phenomena into a cohesive framework, capturing overarching themes and relationships within the data. This iterative approach continued until data saturation was achieved, ensuring a comprehensive understanding of participants’ experiences.

### Ethical Considerations

The study received approval from the institutional review board (IRB) at Rutgers, The State University of New Jersey (IRB# Pro2021001731). At the beginning of each interview, participants were informed about the research procedure and their rights as participants, including the voluntary nature of participation and the right to withdraw at any time. All participants provided voluntary, informed, and written consent to participate in the study and had the interview audio recorded. All recordings were deleted upon transcription. The research team ensured that the transcripts contained no identifying information about the participants and that all data was stored on a passcode-protected drive accessible only to the research team. As a token of appreciation, participants received a US $20 e-gift card upon completing the interview.

## Results

### Overview

Through an analysis of participants’ experiences in providing care services to residents, three key themes emerged: (1) the current state of care workers’ daily responsibilities, (2) care workers’ perspectives on their engagement in technology support, and (3) challenges and labor demands associated with providing technology support. Since this study was conducted in an assisted living setting, we use the term “resident” to refer to the older adults served by the participants. This terminology also aligns with how participants commonly referred to those in their care.

### The Current State of Care Workers’ Daily Responsibilities

#### Multifaceted Job Responsibilities

At the beginning of each interview, participants were asked to describe their job titles, primary responsibilities, and a typical day at work. Despite variations in job titles, all participants reported being actively involved in providing a range of direct care services to residents, extending beyond the generally defined duties associated with each job title. Caregivers, for instance, described a wide array of duties addressing residents’ individual needs holistically, including assistance with meals, hygiene, medication, mobility, and companionship. Participants in medical-care-oriented positions, such as nurses and medication assistants, whose primary responsibilities involved medication administration, condition monitoring, and care plan development, also frequently assisted with personal care tasks, including hygiene and mobility support. Similarly, the administrator, despite primarily handling administrative duties, also engaged directly with residents and their families to ensure their needs were met. Across participants’ narratives, the extensive list of daily tasks highlighted the labor-intensive and multifaceted nature of their work.

I help in assisting condition management...administering medications...monitoring them, and ensuring that they carry out the proper exercises...I also help them with grooming and hygiene needs...household tasks like washing the dishes and cleaning up pots.

Participant 6, caregiver

Medication administration, insulin administration, doctor's visits...Assistance with transfers, ambulation, personal hygiene...On some occasions, nurses would help with showers if we had a shortage of nurses. I am in charge of a lot of responsibilities that change on a daily basis.

Participant 14, nurse

There was a strong consensus among the participants that their primary goal was to provide responsive, individualized care tailored to residents’ needs. Consequently, their daily tasks were not rigidly defined but instead heavily shaped by evolving demands of those they cared for. The fluid nature of their work demanded flexibility and adaptability to provide timely and effective support, requiring them to juggle multiple responsibilities and roles. However, the continuous demands of addressing age-related frailty and the complexities of care sometimes led to experiences of burnout.

In the mornings, people usually call on the system for assistance if they need to. And then they start looking for things to do. We also accompany them if they want us to or if they need supervision. So, it's not like I have a specific role that you carry out every day. I have to switch and perform different roles.

Participant 19, activities assistant

My typical day at our facility was kind of hectic because I had a lot of roles to take on in the facility. That is the administration of medicine, and also daily monitoring and reporting of vital signs. These residents, most of them are old, so they get sick quite often. On a busy day, on average, my day was kind of tiresome because I had to do this work...it was always tough for me.

Participant 13, nurse

Among all these responsibilities, technology support consistently emerged as a significant and recurring task, which we explore in detail in the next section.

#### Tech Support as a Frequent Need of Residents

When initially outlining their responsibilities, participants rarely mentioned providing technology support. However, upon deeper reflection on their daily interactions with residents, in the context of technology use, it became clear that technology is well-integrated into residents’ lives, but often at the condition of their ongoing assistance. Across the board, participants reported varying levels of digital technology use among residents in their facilities, and that many residents frequently relied on their assistance to use these technologies. The most used devices were residents’ personal smartphones and tablets, which they primarily used to access a range of mobile apps. Communication apps, such as WhatsApp, Facebook, and Skype, were frequently used to stay connected with family members and access social networks outside the facilities, as well as to stay informed about news. Additionally, some residents were observed using health and wellness applications, including those for fitness tracking and medication management. A smaller number of participants noted residents using gaming apps and streaming services, such as YouTube. Beyond personal devices, some facilities provided residents with access to shared technological resources, actively encouraging their use. For instance, one facility offered virtual reality equipment to enhance exercise routines, while another maintained a computer lab for residents’ personal use. In many cases, participants noted that residents, whether using personal or facility-provided technology, frequently sought their assistance when encountering difficulties or needing additional guidance.

They mostly use the apps and platforms I mentioned to reconnect with their family members back home. So, these are the most commonly used in the facility...Some use it on their own, while others seek assistance from me and other staff working in the facility.

Participant 5, caregiver

They like to make video calls with their family, like Skype, and use the laptop. Sometimes, we help them out with that. And there are VR fitness oculars that we sometimes use to engage them in physical activities. And they actually also require assistance with using them.

Participant 12, primary caregiver

When explicitly prompted to reflect on the frequency of technology support, participants not only revealed the high occurrence of such tasks but also the high percentage of residents performing them. This suggests that providing technology support becomes an essential part of their daily duties, alongside their other caregiving responsibilities.

(Q: Do residents often ask for instructions on using these technologies? [referring to residents’ phones]) Yeah, most of the time, 80% of time I would say.

Participant 18, medication assistant

(Q: Do residents often ask for instructions [referring to the in-facility computer lab]?) Yeah, I'd say a big percentage of them. They ask for help whenever they need help and instructions.

Participant 13, nurse

### Care Workers’ Perspectives on Their Engagement in Technology Support

#### Care Workers’ Degrees to Accepting a Technology Support Role

As participants reflected more deeply on residents’ technology use and support needs, their narratives revealed varying attitudes toward the boundaries between providing tech support and their assigned job responsibilities. On the one hand, some participants viewed tech support work positively, embracing it as a natural extension of their duties. This perspective was often rooted in the belief that technology assistance represents an emerging and essential need among residents. Given that their responsibilities were already seen as multifaceted, they felt that addressing these needs naturally aligned with their role in providing comprehensive care.

(Q: Do residents help each other with technology?) No, they don’t. Because it’s the staff’s job to help them.

Participant 8, care assistant

When residents have difficulties with technology or ask for help, we were all more than happy to help with computers or cell phones...because it was something for them.

Participant 14, nurse

In contrast, other participants perceived technology support as outside the scope of their assigned responsibilities. While not entirely opposed to assisting residents, they emphasized the challenges associated with incorporating an additional tech support role into their already multifaceted duties and demanding schedules. This burden was further exacerbated by staffing shortages in long-term care facilities. In response, some participants advocated for alternative solutions, such as hiring dedicated technology support professionals, assigning specific staff for tech-related tasks, or implementing resident training programs to promote digital independence and reduce reliance on care workers for technical assistance. These varying perspectives demonstrated the tensions between evolving care demands in the digital age and traditional understandings of caregiving.

Staff is busy. It’s not always feasible to help somebody. They have their own work to do.

Participant 15, nurse

Problems arise due to not having enough staff to show residents how to use it...I know a lot of facilities right now experience a lack of staff. Especially with using technology, I believe it needs to be professional, in some way.

Participant 3, case manager

It would be a nice development if all the facilities can give some kind of sensitization or training to residents on how to operate basic technologies themselves, other than leave the tasks solely to staff.

Participant 4, caregiver

#### Dual Recognition of Technology as Essential and Independent Use as Unachievable

Despite different opinions on whether technology support was a part of their core duties or not, all participants continued to assist residents with technology. This was driven, in part, by a shared acknowledgment that technology use is important to their residents and particularly beneficial in providing the familiar emotional support that participants were unable to offer. As identified by existing studies, one of the major challenges older adults residing in long-term care facilities face is experiences of loneliness and isolation due to reduced opportunities to interact with their families and neighbors [49]. Participants’ narratives also echoed these findings, underlying the prevalence of this issue, particularly among those whose families did not visit regularly. In this case, ICTs served as the primary tool for maintaining residents’ communication with their social networks outside the facilities.

They seek out help regularly, because it's not something they can do without. They have to stay in touch with their loved ones, and they rely on technology to receive daily reminders about their medications.

Participant 16, nurse

A lot of families come in to visit, but some of them don't...Nowadays, Facebook and Skype is probably the best way since technology makes it much easier.

Participant 15, nurse

They would have a better quality of life if they can actively participate using technology to improve their memory and well-being, because technology has its minuses and pluses. But overall, it keeps people connected in such a disconnected world.

Participant 2, case manager

Moreover, there was a collective acknowledgment among participants that the issue related to residents’ heavy reliance on care workers for tech support is inevitable. As revealed by participants, it is simply not possible for residents with heightened age-related physical and cognitive disabilities to use technologies independently. While their deep understanding and empathy for their residents are admirable, it is also crucial to attribute the root cause of this issue not to the inevitable age-related constraints, but to the flawed, existing technology systems that consistently overlook the accessibility needs of older adults [50]. In one facility where a voice assistant was used, the participant demonstrated ongoing difficulties; as such, the device consistently failed to recognize and execute the commands of Spanish-speaking residents. She explicitly attributed her residents’ reliance on staff for tech support to the lack of inclusive technology design, highlighting how language barriers further complicated their ability to navigate digital tools independently.

I think it's normal for someone of their age or physical situation to sometimes require some assistance with the use of technology.

Participant 12, caregiver

Not everybody's able to do it due to memory issues...so we're always there to assist them.

Participant 15, nurse

It's very difficult for bilingual residents to use, because in our facility we have Spanish-speaking residents. They have accents and intonation when speaking English. It's very hard for voice assistants to pick up those particular commands from them. So, there are a lot of errors...Those are the reasons related to the interface of those particular technologies that pose difficulties for them...they seek out help regularly.

Participant 16, nurse

#### Technology Support as a Rewarding Experience

While perceptions toward the role boundaries between caregiving and technology support varied, there is a general acknowledgment that the experience of tech support could be rewarding. For instance, participants shared sentimental stories of helping residents reconnect with a long-lost family member through videoconference or experience the outside world through virtual reality devices. Furthermore, participants' kindness and efforts in providing technology support were reciprocated by residents’ gratitude and positive emotional feedback, allowing participants to have a sense of meaning and fulfillment in their work. By supporting residents with technology-related issues, participants also strengthened trust and fostered positive relationships, creating a more positive work environment.

They do appreciate my assistance with assisting them with their technology.

Participant 4, caregiver

By integrating them with necessary VR applications, we are able to take them back to their favorite places, like going on tours or climbing mountains...While such interventions can take hours...they responded positively. So it wasn't that stressful.

Participant 16, nurse

I was helping a resident establish a full connection to a podcast they were listening to. They needed assistance to get the full connection, and they were very happy...We are there to help all of them, so I am always available to help any resident who needs assistance.

Participant 13, nurse

### Challenges and Labor Demands Associated With Providing Technology Support

#### The Repetitive Burden of Tech Support

Although not all participants explicitly voiced complaints and concerns about the workload associated with providing tech support, a closer examination of their detailed experiences revealed that this role was both physically and emotionally demanding. When prompted to share their observations on the coping strategies residents used to deal with technology-related difficulties, only a few participants mentioned occasional instances of mutual support among residents. In most cases, participants identified that residents relied primarily on staff for assistance, positioning care workers as the first responders to residents’ tech-related requests with the expectation that they would be readily available whenever needed. The high volume and frequency of these demands further exacerbated their already labor-intensive primary responsibilities, contributing to burnout, as outlined in the first section.

I feel like their greatest strategy is the fact that they actually have the staff to rely on. So, whenever they have difficulties and all of that, they have the staff at their disposal to help them out.

Participant 7, care assistant

The only strategy they have is calling for help. So, when they aren’t understanding, you have them yelling your name, trying to call you. So sometimes you can have like five people calling you at the same time. It can be really draining and challenging.

Participant 18, medication assistant

Moreover, the workload was further complicated by the repetitive nature of technology-related requests, which often involved recurring issues. As previously mentioned, residents required the most support with emerging digital technologies, particularly mobile apps on their phones and tablets. Modern apps often require users to navigate multistep interactions, manage various settings, and adapt to frequent updates to fully utilize their features. Consequently, assisting residents often involves step-by-step navigation and detailed instructions. However, the initial guidance efforts seldom led to meaningful learning or skill acquisition among residents, leading to repeated interventions for the same issues. As a result, tech support work often became a repetitive task, requiring significant patience, time, and emotional energy, which further strains participants’ already demanding roles.

You just try to open the apps, see the updates, and then just explain to them what the problem is and show them how they’ll go about it. Then the next time they are in the same situation.

Participant 19, care assistant

Initially, he was unsure of how to use it (an app that tracks residents’ movement). I explained that when he wakes up, he needs to open the app, place the phone either on his body or hold it in his hand...He didn't fully understand on the first day, so I had to repeat the instructions multiple times.

Participant 9, care assistant

#### Additional Responsibilities Associated With a Tech Support Role

In addition to the taxing and multifaceted nature of juggling multiple roles and responsibilities, their experiences also revealed that “tech support” encompasses far more than simply addressing technical difficulties. Instead, it often required participants to take on multiple responsibilities, often involving complex coordination tasks. Many participants found themselves acting as coordinators, facilitating communications between residents and third parties, such as families and medical providers. These coordinating tasks included setting up the devices, coordinating schedules with various external parties, and even securing permissions for communication when needed. These responsibilities were particularly pronounced for participants assisting residents experiencing cognitive impairments.

I always double-check with the family if it’s okay to [call] or no, because you don’t know what they’re gonna open up and you know viruses and everything else on the internet.

Participant 15, nurse

We tend to help them do that because some of them don't get to have their telephones with them. So, we tend to call their families for them on a weekly basis or, in special cases, like the woman (with dementia and only remember her child at times) that wanted to see her child at the time. So, we tend to communicate to the families for them.

Participant 10, care assistant

Several facilities that offered communal technology resources and devices for residents actively promoted their use to enhance residents’ health and social engagement. However, these technologies were rarely used by residents without assistance from participants. In facilities where these technology resources were more established, there were formal guidelines regulating residents’ use of technology. Participants’ narratives suggest that such regulation stems from the facility’s concern about potential damage to the high-cost devices. As a result, participants were directly assigned supervisory responsibilities by their employers to ensure the proper functioning, maintenance, and safe use of these devices. Additionally, while the purpose of technologies such as medicine dispensaries is to facilitate residents’ disease self-management, their lack of technology proficiency also led to heightened concerns among participants about the potential health hazards resulting from technology misuse. Consequently, participants voluntarily took on supervisory roles to monitor residents’ technology use.

In our facility, we have rules because if residents are allowed to use this technology on their own, they may mess things up. So, we guide them to ensure maximum utilization of the technology.

Participant 13, nurse

This digital medication dispenser, there was a day a resident who is on medication forgot to take her medication. So, she was having doubts since she had missed a dose...I just had to monitor her to ensure that she made use of the dispenser in my presence.

Participant 20, administrator

Moreover, intertwined with their coordination role, participants often had to advocate for technology use among residents who avoided it for various reasons, particularly in facilities with communal technology resources. Aligning with prior research on older adults’ reduced interest in technology adoption [11], participants observed varying degrees of technology hesitation, especially among the oldest residents. Despite well-intentioned efforts to introduce mobile apps and other emerging technologies to enhance residents’ health and social well-being, these efforts were sometimes met with dismissiveness or resistance. Encouraging reluctant residents to engage with technology required patience, empathy, and careful navigation of their moods to foster a supportive and encouraging environment.

Most of them are a bit older than I think, and for them to embrace the technology, they need so much convincing.

Participant 19, activity assistant

I had an encounter with one of the residents who was basically not in the mood because...she didn't want to actually use the app...I tried to convince her that it would be beneficial for her to try it.

Participant 7, care assistant

#### Emotional Dimension of Tech Support

Navigating technology later in life without prior exposure to digital learning experience can be emotionally challenging [51,52], particularly for older adults who wish to use technology but struggle to do so. Participants found these difficulties often triggered a range of negative emotions among residents, including frustrations, confusion, and mood swings, which created challenges in their daily work. As a result, participants were responsible not only for addressing technical problems but also for managing residents’ emotional responses while using technologies. To de-escalate these situations, participants used various strategies, such as providing encouragement, offering empathetic guidance, and maintaining patient and supportive communication.

What I've observed is that it's become difficult for some of them; they get depressed, or they feel less motivated because they are trying to access these technological platforms, but it's hard for them. Whenever I find them in these kinds of situations where they feel down or their mood is low, I initiate interactive conversations and some fun activities.

Participant 5, caregiver

They start with panic because something they really need is not working...So the interaction usually begins in a panic mode. After interacting with them, I assured them that we will take care of it and fix the issue as soon as possible. Then they calm down.

Participant 6, caregiver

Defensiveness was a particularly common emotional response among residents who highly valued their independence and believed they possessed sufficient knowledge. This often manifested as stubbornness or a reluctance to seek help. While frequent tech support requests could overwhelm participants’ already strained schedules, residents’ intentional avoidance of seeking help posed a different challenge. This was especially true for those managing communal technological devices, as they often had to resolve issues caused by residents’ mishandling. To manage these situations, participants adopted proactive and sensitive approaches when offering assistance, ensuring that residents felt their independence was respected while still receiving the support they needed.

Some of these residents are stubborn...because they think they know, but they don't know. That's why it's important for them to seek help. Some of them are actually stubborn because they always assume they know things, but whenever they're trying to apply, they always mess up.

Participant 13, nurse

Most of the time when they come around...“I need assistance with this. I need to check this, I need to check that, and can you please help?”...If it's someone who couldn't act fast or you know that this person needs assistance, you just ask the person, Can I help you? Can I help you to do this?

Participant 11, care assistant

In turn, managing residents’ emotions negatively impacted participants' own emotional well-being and can be mentally draining [53]. Yet, their emotional labor often goes unrecognized by their employers and even by the participants themselves, overshadowed by the deep emotional bonds they share with residents [54]. Correspondingly, in this study, while participants did not explicitly describe technology support work as emotionally burdensome, the constant need to navigate residents’ frustrations with patience and sensitivity, alongside physical tasks, suggested that such work takes an emotional toll. Participants’ narratives made it clear that to provide effective and satisfying technology support, they frequently needed to regulate their own emotions, even in the face of challenging behaviors.

One of the most important things in working in assisted living is that you have to always be calm, even when you are not pleased with some behaviors of the residents; you have to be the one who has to be calm.

Participant 7, care assistant

## Discussion

### Overview

Recent studies on technology use in long-term care facilities revealed the frequent need for care workers to provide technology assistance [21,22]. Nevertheless, research examining care workers’ experiences with tech support remains limited. This underscores the need for a deeper understanding of their evolving role in the digital age. This study specifically focuses on the perspectives and experiences of care workers in assisting residents with technology in assisted living settings. In what follows, we discuss implications for better recognizing, supporting, and including care workers in the integration of technology in long-term care.

### Increase Recognition of Labor Associated With a Tech Support Role

Our findings highlight that caregivers’ technology support extends beyond assisting with devices, encompassing various roles and tasks generated by one seemingly singular tech support role. Echoing existing studies [21], tech support often leads to various coordination activities involving external parties, such as residents’ families and health providers. Additionally, our studies uniquely highlight the supervisory responsibilities associated with maintaining and ensuring the safe use of technology devices in care facilities. This burden is particularly pronounced among participants in facilities that implement on-site technological solutions, contributing to workplace stress. As participants indicated, the potential damage to high-cost in-facility devices or residents’ misuse of technology can be perceived as a failure in their professional practice. Such pressure, in turn, can compel them to take on even more supervisory and support tasks to mitigate risks, ultimately creating a relentless cycle of labor. As vulnerable workers, even minor perceived failures can overshadow their significant daily contributions, highlighting the urgent need for greater recognition of their labor input.

This study also highlights the emotionally challenging nature of tech support in caregiving. Research increasingly acknowledges the emotional labor inherent in caregiving, defined as the regulation of one’s emotions to align with expected professional expressions, even when they conflict with personal feelings [33,53]. Consistent with prior studies [13,51], our participants described how older adults’ struggles with technology often trigger frustration, confusion, and emotional outbursts. Consequently, caregivers frequently become the direct target of these frustrations, absorbing residents’ emotional distress while striving to offer patient and compassionate support.

Despite being an integral part of resident care, these additional layers of labor associated with a tech support role remain largely unrecognized. Job descriptions and verbal accounts of caregiving roles often fail to acknowledge the full extent of tech support responsibilities. As previous research noted, care workers’ dedication to their professional identity should not justify the normalization of excessive labor [23,28,29]. The continued lack of recognition perpetuates the devaluation of caregiving work [28]. Therefore, given the rapid digitalization of the care sector, this study emphasizes the need for ongoing research and policy interventions to recognize the physical and emotional investments made by care workers in providing tech support and how these responsibilities contribute to their already demanding workloads. Additionally, employers must implement stronger support systems, such as on-site mental health services and training, and recognize this labor through improved compensation and workplace policies.

### Technology Use, Design, and Implementation in Long-Term Care Needs Ongoing Careful Evaluation

We found a wide range of technologies are used in assisted living, including residents' personal devices and assistive technologies provided by the facilities. Participants also expressed an overall positive attitude toward residents' use of technology and their willingness to offer support. This willingness stemmed not only from residents’ requests but also from participants’ genuine belief that technology enhances the quality of life for residents. These findings align with the broader discourse and research on technology in long-term care, where its potential to improve the social and physical well-being of older adults has been widely acknowledged [6,7].

By highlighting how technological innovations introduce new responsibilities for care workers, this study calls for a more nuanced perspective in the ongoing discourse and research on the use of technology and caregiving in long-term care. While technologies can enhance service delivery, support aging independence, and assist care workers, their successful implementation should not be discussed in isolation from the labor of care workers. Too often, the discussions around technology in long-term care focus on the potential for automation to replace routine aspects of caregiving, which risks further devaluing care work [55]. Yet, as this study and others have shown [21], the success of residents’ technology use and the implementation of new devices in facilities remain heavily dependent on the support of care workers. Future studies should evaluate the effectiveness of technology use in long-term care by centering care workers’ perspectives on both the benefits and shortcomings these innovations introduce.

Moreover, the significant challenges older residents experienced when using these devices and their reliance on others for support, as identified by participants, highlight the persistent digital divide and the lack of age-inclusive design thinking in current technology systems [50,56]. While some may argue that such challenges are unique to the current cohort of older adults, who may have had limited opportunities to engage with technology earlier in life, other challenges, particularly those associated with age-related health conditions such as cognitive impairment, will remain relevant going forward. These findings further demonstrate the lack of inclusivity in existing technology design and the urgent need for improvement.

Existing studies on the technology use of older adults have advocated for a user-centered approach, emphasizing the importance of involving older adults in the design process [57-59]. Building on this, our study recommends the inclusion of care workers in technology design to integrate their expertise and acknowledge their central role in supporting older adults. By involving care workers, we can create more inclusive and practical care-based technologies that address the challenges faced by both older adults and their caregivers. Moreover, this collaborative approach ensures that technological advancements do not come at the expense of frontline care workers, reinforcing the need to value their labor rather than further marginalizing it.

### Amplify Care Workers’ Agency in Defining and Navigating the Work Boundaries

Consistent with previous research [29,60], this study demonstrates that care workers’ job roles are often ambiguously defined and shaped by the evolving needs of residents. Then, the added responsibility of providing tech support further complicated their work [29,30]. While some participants attempted to distinguish their caregiving duties from tech support, their ongoing assistance with residents’ technology needs blurred these boundaries over time. This role expansion, driven by their dedication to resident well-being, contributed to an increased workload and emotional exhaustion, aligning with prior research that links caregiving to burnout [61].

However, given the dynamic nature of person-centered care in assisted living, rigidly defining caregiving responsibilities may not be the most effective approach. Indeed, a degree of flexibility is beneficial, as it allows care workers to respond adaptively to residents’ needs while reducing workplace strain [60,61]. Therefore, rather than imposing strict role definitions, we emphasize the importance of empowering care workers to define their own work boundaries within a supportive environment [60]. In practice, encouraging open discussions with peers and supervisors will help establish and adjust these boundaries as needed. Lastly, we recommend that employers acknowledge the additional labor associated with less-recognized tasks, such as tech support, and create spaces for care workers to reflect on these responsibilities. Practical steps include offering paid workshops, professional development opportunities, and structured communication channels to foster collaboration and ensure that care workers feel supported in balancing their evolving responsibilities while maintaining their well-being.

### Limitations

Our findings must be considered in light of several limitations. First, our sample consisted mainly of younger care workers who did not report experiencing tech-related difficulties while assisting residents. Given that the US caretaking workforce is aging, with a median age of 47 years [62], future research should examine how age-related factors influence care workers’ ability to provide tech support. Second, although we did not find differences in experiences across job titles, this may be because our analysis primarily focused on general care-support tasks that remain consistent across caregiving roles. Moreover, the small sample size within each job category might have limited the feasibility of comparative analysis. Future studies should include a larger sample to explore potential variations in experiences across different caregiving positions. Third, all participants were based in the United States, meaning the findings primarily reflect Western perspectives. Cross-cultural comparisons can provide a more comprehensive understanding of how care workers use technology support. Fourth, the study relied on self-reported experiences, which may be subject to bias. Participants might have underreported difficulties or overemphasized their ability to provide tech support, potentially skewing the findings. Future research could incorporate diverse methods to capture a more objective understanding of the challenges faced by care workers.

### Conclusions

Given the rise in technology adoption among older adults and in long-term residential facilities, this study explored the perspectives and experiences of care workers in providing technology support to residents. We identified the nuanced physical and emotional labor involved in technology support, which has become a core yet often unrecognized aspect of caregiving. Additionally, the varying perspectives of care workers on their technology support roles highlight ongoing tensions in work expectations within long-term care. Based on these findings, we advocate for greater recognition of care workers’ efforts through recommendations for further research, improved workplace policies, and enhanced program design in long-term care.

## Data Availability

Data will be made available on reasonable request.

## Appendix

Multimedia Appendix 1 Semistructured interview guide.

## Abbreviations

ICT: information and communication technology
IRB: institutional review board
mHealth: mobile health
