# Supplementary material for: Providing Tech Support as Care Work Among Care Workers in Assisted Living Facilities: Qualitative Interview Study
Source: JMIR Aging. 2026 Feb 26;9:e80272. doi: 10.2196/80272 (PMC12982957; doi:10.2196/80272)
Supplement: Multimedia Appendix 1 [file aging_v9i1e80272_app1.docx]

**Semi-Structured Interview Guide**

1. To start, could you tell me a little bit more about the facility? For example, how many residents and staff there are, what the environment is like, or anything else you think is important to understand the setting.
2. What about your role? Could you describe your role and its primary responsibilities?
   1. Could you describe what a typical day looks like for you at this facility?
   2. On average, what percentage of your work time involves direct interaction with the residents?
3. When you think specifically about your work with residents, what kinds of tasks do you typically do when you interact with them?
   1. How often do you find yourself doing these tasks?
   2. On an average workday, about how many residents do you interact with one-on-one?
4. Could you share one or two recent interactions with a resident that stand out in your memory?
   1. Is this type of interaction something you do frequently with that resident?
   2. Do you also do this for other residents?
5. Could you tell me your overall impression of the residents' technology use in your facility?
   1. What kinds of technologies have you observed residents using, and for what purposes (for example, recreational, communication, or health-related activities)?
   2. Are there any specific types of technology that residents are required to use in your facility?
   3. Do residents use these technologies independently?
   4. Are there any technologies you believe residents would benefit from but are not currently using?
   5. Do you have any suggestions on how to improve residents’ technology use at your facility?
6. Do you help residents with technology? If so, could you describe a recent interaction you had with a resident that involved the use of technology?
   1. Who typically initiated this interaction?
   2. Could you describe to me how the residents responded to the interaction?
   3. Is this type of assistance something you have also provided to other residents? If so, could you provide more information about those interactions?
   4. How often do you find yourself providing this kind of technology-related support?
   5. Do residents ever support one another with technology?
7. How comfortable are you with using technology?
   1. Have you encountered any challenges while providing technology support to residents?
   2. Are there any types of technology that you think the staff has difficulty using?
   3. Are there any specific technologies that staff members need to use to perform their jobs? If so, how would you describe your experience using them? Have you encountered any challenges? And how do you overcome those challenges?
   4. Do you have any suggestions for improving your overall work experience at the facility?
8. More broadly, do you have any suggestions for improving your overall work experience at the facility? Before we wrap up, is there anything else you would like to share?
